# Lytic EBV infection investigated by detection of Soluble Epstein-Barr virus ZEBRA in the serum of patients with PTLD

**DOI:** 10.1038/s41598-017-09798-7

**Published:** 2017-09-05

**Authors:** Mohammed Habib, Marlyse Buisson, Julien Lupo, Felix Agbalika, Gérard Socié, Raphaele Germi, Monique Baccard, Berthe-Marie Imbert-Marcille, Jacques Dantal, Patrice Morand, Emmanuel Drouet

**Affiliations:** 1 Institut de Biologie Structurale (IBS), Université Grenoble Alpes, CEA, CNRS, Grenoble, France; 2 Unit of Virology, University, Hospital, Université Grenoble Alpes, Grenoble, France; 3 Unit of Virology, Hôpital Saint-Louis, AP-HP, Université Paris-Diderot, Paris, France; 4 Unit of Clinical Hematology, Hôpital Saint-Louis, AP-HP, Université Paris-Diderot, Paris, France; 5 Unit of Virology, University Hospital, Université de Nantes, Nantes, France; 6 Unit of Nephrology & Clinical Immunology, University Hospital, Université de Nantes, Nantes, France

## Abstract

The ZEBRA protein (encoded by the BZLF1 gene), is the major transcription factor of EBV, expressed upon EBV lytic cycle activation. Several studies highlighted the critical role of EBV lytic infection as a risk factor for lymphoproliferative disorders like post-transplant lymphoproliferative disease (PTLD). Here, we use an antigen-capture ELISA assay specifically designed to detecting the circulating soluble ZEBRA (sZEBRA) in serum samples (threshold value determined at 40ng/mL). We retrospectively investigated a population of 66 transplanted patients comprising 35 PTLD. All the samples from a control population (30 EBV-seronegative subjects and 25 immunocompetent individuals with EBV serological reactivation), classified as sZEBRA < 40ng/mL were assigned as negative. At PTLD diagnosis, EBV genome (quantified by qPCR with EBV DNA>200 copies/mL) and sZEBRA were detectable in 51% and 60% of cases, respectively. In the patients who developed a pathologically-confirmed PTLD, the mean sZEBRA value in cases, was 399 ng/mL +/− 141 *versus* 53ng/mL +/− 7 in patients who did not (p  < 0,001). This is the first report relating to the detection of the circulating ZEBRA in serum specimens, as well as the first analysis dealing with the lytic cycle of EBV in PTLD patients with this new biomarker.

## Introduction

Epstein–Barr virus (EBV) is a human herpesvirus that causes infectious mononucleosis. It is also associated with the development of certain malignancies, including African Burkitt lymphomas (BL), B-cell lymphomas of immunocompromised patients, nasopharyngeal carcinomas (NPC), Hodgkin’s disease, and, occasionally, with T-cell lymphomas and gastric cancers^1^. Like all herpesviruses, EBV can infect cells in either latent or lytic forms. Latent infection occurs in memory B cells, allowing the virus to evade the host immune response and to persist indefinitely within humans^2^. Regardless of cell type, all EBV-associated malignancies largely consist of latently infected cells in which EBV-encoded transforming proteins and non-coding RNAs are expressed^2^. Immunocompromised individuals present a lack of T-cell control which ultimately favors the expansion of B-cell clones that are infected and immortalized. These cells may also gain additional genetic lesions which causes oligoclonality and, ultimately, monoclonality of the B-cell proliferation. The status of Immune-compromised patients after Hematopoietic Stem Cell transplantation (HSCT) or solid organ transplantation may destroy the normal balance between proliferative capacity of latently infected B-cell and also the EBV-specific T-cell response. Therefore, the increased number of latently infected B-cells may lead to aggressive post-transplant lymphoproliferative disorders or PTLD^3^. Currently, the PTLD etiology is still unclear, although 60–80% of cases were associated with EBV infection, which has been proposed as a major factor contributing to PTLD development^3, 4^. While the risk of lymphoma development after organ transplantation is about 20–120% higher than in the normal population, it is still highly challenging to predict which transplant recipients will ultimately develop PTLD^5^. The PTLD occurrence is generally preceded by increased EBV viral loads in the blood related to a so-called “viral reactivation” and by an increased number of infected B-cells^6, 7^. In PTLD patients, the increased EBV load in mononuclear cells (PBMCs) of the peripheral blood can be accounted for by an increased number of circulating EBV-positive cells. These cells represent memory B-cells rather than proliferating lymphoblast cells, and thereby resemble latently-infected resting B-cells, with a restricted set of EBV latency genes^2, 8, 9^. Indeed, high circulating EBV levels seem to reflect the tumor burden that can be monitored during treatment^10–13^. Several experiments highlighted the role of immediate-early (IE) lytic viral protein expression in the lymphomagenesis in immunocompromised mice^14^. Other studies pointed out the crucial role of lytic EBV infection in the development of B-cell lymphomas in thymic tissue-reconstituted mice or cord blood humanized mice^15–17^. The ZEBRA protein encoded by the BZLF1 gene, is the major transcription factor of EBV, expressed upon EBV lytic cycle activation, while directly regulating the expression of a viral gene set^18, 19^. Moreover, ZEBRA exhibits a highly peculiar trait in that it preferentially binds to a subset of CpG-methylated, rather than unmethylated, ZEBRA-responsive elements^18^. As ZEBRA was reported able to activate host cellular genes^20–24^, the reactivation of EBV lytic cycle was also shown to contribute to the growth of latently-infected cells^14, 16^ and this, by promoting the release of paracrine B-cell growth factors^25^. All in all, all these studies showed that the presence of a limited number of lytically infected cells may enhance tumor growth through release of growth factors and immunosuppressive cytokines^14, 15, 25^.

The current report describes the detection and quantification of the circulating ZEBRA protein in serum samples from transplanted patients. Most surprisingly, our research revealed that this protein, usually found in the nucleus of EBV-infected cells, was also detected in the serum of patients suffering from EBV-associated diseases.

## Materials and Methods

### Patients and samples

We studied 66 transplanted patients retrospectively selected patients from different hospitals in France: (i) 23 PTLD subjects were selected from a database of patients (DIVAT clinical prospective cohort (www.divat.fr, CNIL 891735) having received kidney transplants at the University Hospital of Nantes between 2003 and 2013; (ii) 22 subjects were additionally selected from a registry of patients (including six PTLD) having received solid-organ transplants (lung and kidney) at the University Hospital of Grenoble-Alpes between 2010 and 2013. In addition, 21 HSCT patients (including six PTLD) were enrolled at Saint-Louis Hospital (APHP, Paris) between 2010 and 2012. All patients were monitored by means of routine viral testing, resulting in 322 serum specimens. The “Cases” were transplant recipients who developed pathologically-confirmed PTLD, with at least two stored serum specimens available. PTLD diagnosis was based on examining histological material obtained by either open biopsy or core needle biopsy, with lesions classified according to the WHO Classification of tumors^5, 26^. Association with EBV was confirmed by *in situ* staining for EBER. For each case, we identified a “prediagnostic” specimen taken as close as possible to the 6-month pre-transplant period, although several specimens were taken at different intervals, depending on specimen availability. The second specimen, namely the diagnostic one, was obtained as close as possible to the PTLD diagnosis. The “Controls” were transplant recipients free of PTLD. Finally, the sZEBRA quantification was validated using an additional control population of 55 subjects, divided in two groups: (i) 30 EBV-seronegative subjects; (ii) 25 immunocompetent individuals with EBV serological reactivation (elevated anti-EBV IgG levels >640 UA/mL by means of the Enzygnost anti-EBV/IgG Siemens Healthcare Diagnostics, Marburg, Germany Siemens test), as described earlier^27^. All serum samples available were decomplemented, collected, then catalogued, and stored at −80° C at the University Hospital of Grenoble-Alpes.

### Quantification of soluble ZEBRA by means of antigen-capture ELISA and neutralization test

An antigen-capture ELISA method was developed specifically for measuring sZEBRA in serum samples, using the IgG2a mAb AZ125 as capture antibody, according to patent application PCT/FR2012/052790^28^. Firstly, the AZ125 mAb was coated onto microplate wells. Following incubation, the plates were washed and then blocked in PBS-gelatin (PBS-G). Serum samples (initially tested at different dilutions of 1/10, 1/50, and 1/100 in PBS-G) or the recombinant protein (r-ZEBRA, see supporting information) were incubated at room temperature (RT) for 1 hour (h). Next, the samples were incubated with biotinylated IgG1 mAb AZ130 detection antibody (see supporting information). The last step consisted of adding avidin-horseradish peroxidise, thereby generating a signal for detection. The optical density (OD) was measured at 450nm (630nm as reference) by means of an Organon Teknika Microwell system (Reader 230s, Germany) (Fig. S1). A standard curve was obtained based on serial dilutions of r-ZEBRA in Phosphate Buffer Saline pH 7.4, ranging from 1ng/mL to 250ng/mL. The results were expressed as concentrations of ZEBRA (ng/mL) extrapolated from the standard curve. In view of determining the detection specificity, three positive serum samples were submitted to a neutralization test (Fig. S3).

### EBV DNA quantification in serum

EBV DNA load (DNA-emia) was measured by qPCR in serum samples as described elsewhere^29^. Briefly, DNA was isolated from 200 microliters of serum using the NucliSens EasyMag automated platform (BioMérieux, Marcy L’Etoile, France). Amplification was conducted by means of the commercially-available EBV R-gene quantification kit (BioMérieux) on the LightCycler 480 platform. EBV DNA load measurements were expressed as copies/mL (limit of detection = 200 copies/mL).

### Statistical analysis

Statistical analysis was performed using GraphPad Instat software, Version 3.05 (Graphpad, San Diego, United states). For univariate analysis, the Chi-squared test was used to assess the association between categorical variables. For correlation Pearson’s test, the R^2^ coefficient was calculated. All tests were two-sided, with a p-value <0.05 considered statistically significant.

### Ethics statement

An informed consent was obtained from all transplant patients. Donor and recipient data were extracted from the DIVAT clinical prospective cohort (www.divat.fr, CNIL Nr 891735 version 2, August 2004). Codes were used to ensure donor and recipient anonymity and blinded testing. The data were computerized in real time as well as at each transplant anniversary. The quality of the DIVAT data bank was validated by an annual cross-center audit, systematic verification during data entry and a weekly automatic report on the identification of incoherencies between parameters. No organs/tissues were procured from prisoners. The Unit of Clinical Hematology (University Hospital Saint-Louis, AP-HP, Université Paris-Diderot), the Unit of Nephrology & Clinical Immunology (University Hospital, Université de Nantes, Nantes, France) provided the serum samples from transplant patients. All methods were carried out in accordance with relevant guidelines and regulations. All experimental protocols were approved by the licensing committee of Institut de Biologie Structurale.

## Results

### Determination of the ZEBRA antigen-capture ELISA characteristics

The two mAbs AZ125 and AZ130 specific for ZEBRA protein were employed in a sandwich ELISA throughout the whole procedure for quantifying captured sZEBRA (Fig. S1). Based on recently published results pertaining to a related patent^28^, significant improvements were made to increase the sensitivity and robustness of the sZEBRA assay. Under these conditions, the technique’s sensitivity was estimated at 2 ng/mL, with variation coefficients ranging from 0.2–18.5% depending of the concentration. The signal and antigen response curve was linear from 15 to 200ng/mL. For determining the limit of detection (LOD), the OD_450_ (and OD_630_ as reference) of negative controls was measured three times independently (mean = 0.14 – Standard deviation (SD) = 0.06). LOD was calculated at 0.35 (*i.e*., mean + 3SD), with a threshold value determined at 40ng/mL. Given these conditions, although all EBV-seronegative sera displayed an OD_450_ value below the LOD (Fig. S2), the optimal serum dilution to maximize the assay’s sensitivity and reproducibility was fixed at 1/10 for the rest of the study. The serum samples of 25 immunocompetent patients with elevated anti-EBV IgG levels were then assessed using this assay, with none exhibiting OD_450_ values above the cut-off value and only one sample reaching the LOD threshold (Fig. S2). Consequently, these samples with sZEBRA < 40ng/mL were assigned as negative.

### Transplant patient characteristics

Table 1 summarizes the 66 transplant patient characteristics. In regards with EBV pretransplant serostatus, cases were less often to be EBV seropositive than controls (61% *versus* 92%). PTLD developed at a median of 2.5, 3.5, and 72 months for patients with HSC, lung, and kidney transplantation, respectively (Fig. S4). The majority of PTLD tumors were monomorphic B-cell lymphomas and EBV-positive. Nine patients experienced atypical PTLD forms (3 Hodgkin’s lymphomas, 2 T-cell lymphomas, 2 Burkitt’s lymphomas, and 2 mantle cell lymphomas)(Table 2).

**Table 1:** Characteristics of the 66 transplant recipients and EBV reactivation (measured by qPCR and by sZEBRA).

	Subjects with PTLD [cases] = 35		Subjects without PTLD [controls] N = 31	
Female, n (%)	12 (34)		10 (32)	
Mean Age at transplant (range)	47 (12–69)		48 (8–67)	
**Organ type**, **n** (**%**)				
Kidney	26		3	
Lung	3		13	
HSC	6		15	
EBV infection, n (%)	qPCR^#^ 18/35 (51)	sZEBRA^##^21/35 (60)	qPCR 4/31 (13)	sZEBRA 5*/31 (16)
**High titers of anti-ZEBRA IgG antibodies (>10,000) n (%)	12/35 (34)		14/31 (45)	

^#^Detection threshold at 200 copies/mL - ^##^Detection threshold at 40 ng/mL.

*Among the five sZEBRA-positive patients, three experienced a primary CMV infection with viral syndrome. The two other sZEBRA-positive patients (HSC transplants) deceased of acute leukemia. The IgG anti-ZEBRA antibodies were titrated by ELISA as previously described^59^. All the serum samples tested were diluted at the dilution 1/1,000. The titres were defined based on the highest dilution at which the OD of a serum sample significantly differed from that of negative serum sample. Titers are given in the reciprocal of the dilution factor.

**Table 2:** Characteristics of the 35 transplant recipients with PTLD.

Patient	Age/sex	Transplanted organ	PTLD Histological subtype	EBV Primary infection	PTLD	sZEBRA^#^	EBV load^##^	Anti-ZEBRA IgG (titers)	Current status
#1B	M/45	Kidney	Mantle Lymphoma	No	Early onset	430	<200	20,000	Alive
#2M	M/66	Kidney	Mantle Lymphoma	No	Early onset	<40	<200	1,000	Alive
#3J	F/67	Kidney	B-cell Lymphoma	No	Early onset	<40	19,000	<1,000	Dead
#4P	M/66	Kidney	B-cell Lymphoma	Yes	Early onset	50	30,000	<1,000	Dead
#5E	F/38	Kidney	B-cell Lymphoma	Yes	Early onset	<40	2,000	<1,000	Alive
#6Y	M/49	Kidney	B-cell Lymphoma	Yes	Early onset	<40	11,000	<1,000	Dead
#7E	F/36	Kidney	B-cell Lymphoma	Yes	Early onset	80	13,000	5,000	Alive
#8K	M/22	Lung	B-cell Lymphoma	?	Early onset	50	43,000	10,000	Alive
#9T	M/48	Lung	B-cell Lymphoma	?	Early onset	84	520	5,000	Dead
#10P	M/12	Lung	B-cell Lymphoma	Yes	Early onset	<40	<200	<1,000	Alive
#11M	F/50	HSC	B-cell Lymphoma	No	Early onset	3700	34,000	5,000	Dead
#12B	M/43	HSC	B-cell Lymphoma	Yes	Early onset	1200	130,000	10,000	Alive
#13A	M/60	HSC	B-cell Lymphoma	No	Early onset	1900	5,000	10,000	Alive
#14A	F/37	HSC	B-cell Lymphoma	Yes	Early onset	1200	1,200	>20,000	Alive
#15A	M/56	HSC	B-cell Lymphoma	No	Early onset	1400	800	1,000	Alive
#16A	M/65	HSC	B-cell Lymphoma	No	Early onset	2300	1,600	1,000	Alive
#17B	M/56	Kidney	B-cell Lymphoma	No	Late onset	<40	<200	10,000	Alive
#18B	F/69	Kidney	Hodgkin Lymphoma	?	Late onset	<40	<200	5,000	Dead
#19B	M/21	Kidney	Burkitt Lymphoma	Yes	Late onset	50	<200	10,000	Dead
#20L	M/39	Kidney	B-cell Lymphoma*	No	Late onset	<40	<200	<1,000	Alive
#21M	M/42	Kidney	B-cell Lymphoma	No	Late onset	50	600	20,000	Dead
#22O	F/52	Kidney	B-cell Lymphoma	No	Late onset	60	5,400	20,000	Dead
#23B	F/59	Kidney	B-cell Lymphoma*	No	Late onset	110	<200	5,000	Alive
#24G	M/64	Kidney	B-cell Lymphoma*	?	Late onset	<40	<200	5,000	Alive
#25A	M/37	Kidney	B-cell Lymphoma*	No	Late onset	<40	<200	5,000	Alive
#26V	M/65	Kidney	Burkitt lymphoma	No	Late onset	<40	<200	1,000	Dead
#27E	F/41	Kidney	B-cell Lymphoma*	?	Late onset	108	<200	10,000	Alive
#28S	M/66	Kidney	T-cell Lymphoma	No	Late onset	61	<200	1,000	Dead
#29K	M/34	Kidney	Hodgkin Lymphoma	?	Late onset	<40	500	1,000	Alive
#30C	M/57	Kidney	Hodgkin Lymphoma	?	Late onset	50	1,700	10,000	Alive
#31M	F/48	Kidney	B-cell Lymphoma*	?	Late onset	50	<200	1,000	Alive
#32B	F/48	Kidney	B-cell Lymphoma*	?	Late onset	55	<200	10,000	Dead
#33F	F/52	Kidney	B-cell Lymphoma	?	Late onset	60	6,500	1,000	Dead
#34M	M/42	Kidney	B-cell Lymphoma	?	Late onset	<40	<200	5,000	Alive
#35P	M/58	Kidney	T-cell Lymphoma	?	Late onset	<40	<200	1,000	Alive

*EBV-negative PTLD.

^#^Detection threshold at 40 ng/mL. ^##^Detection threshold at 200 copies/mL.

### EBV infection in transplant patients (cases and controls)

**Figure 1:**
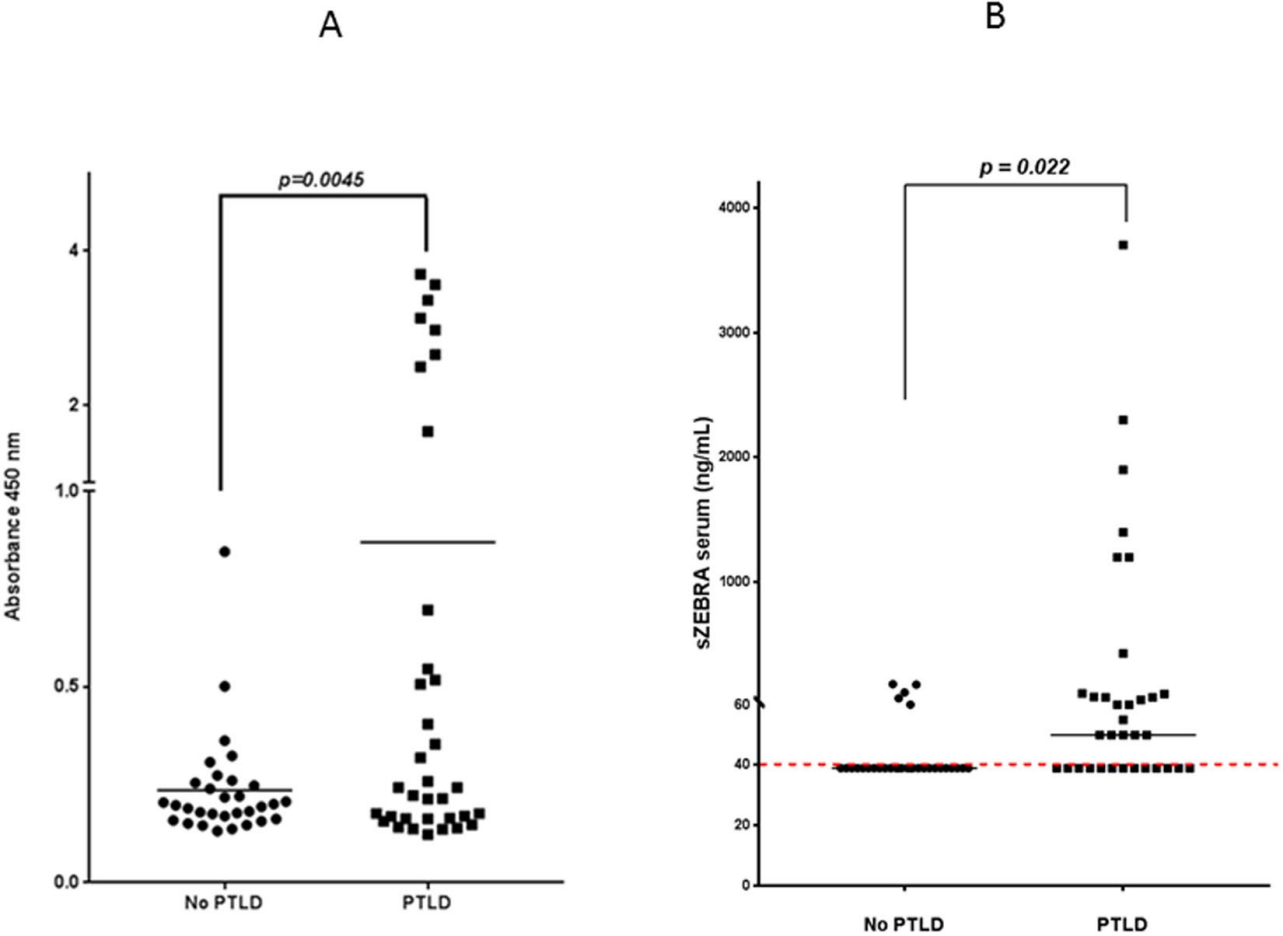
Quantification of sZEBRA at diagnosis of PTLD, in serum samples from the 66 transplant patients (cases and controls). The LOD corresponds to an OD_450_ value of 0.35 (Figure 1A) and to 40 ng/mL (Fig. 1B). The mean OD_450_ values (and levels in ng/mL of sZEBRA) were significantly higher (p < 0.0045 − p = 0.022, respectively) in PTLD cases than in control patients, regardless of the organ transplant type. Specimens corresponding to an OD>3 were diluted at 1/100. The median is indicated by a bar.

**Figure 2:**
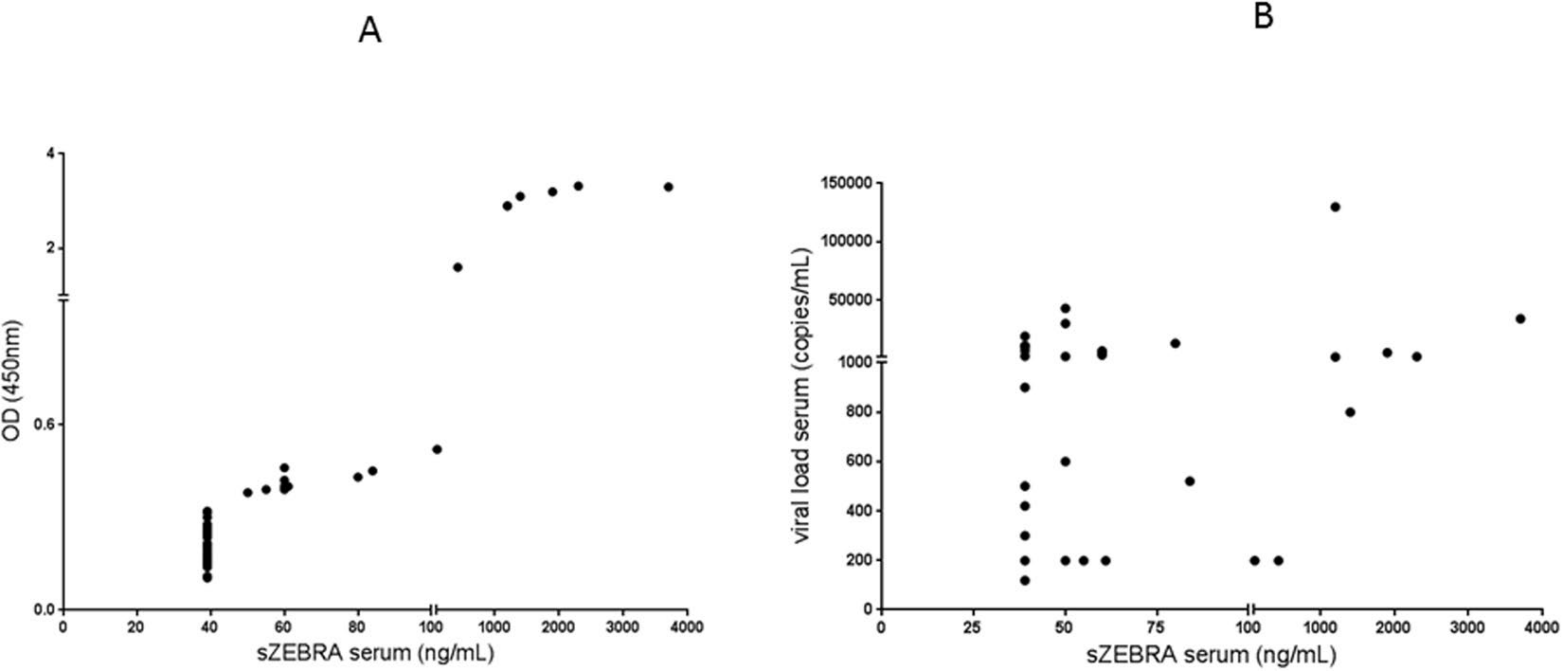
Correlative study between sZEBRA, OD_450_ values and Viral load measured by PCR: Fig. 2A shows the high correlation between OD_450_ values and sZEBRA (p < 0.0001). Figure 2B shows the absence of correlation between sZEBRA (ng/mL) and EBV viral load measured by PCR (copies/mL) (p = 0.098).

**Figure 3:**
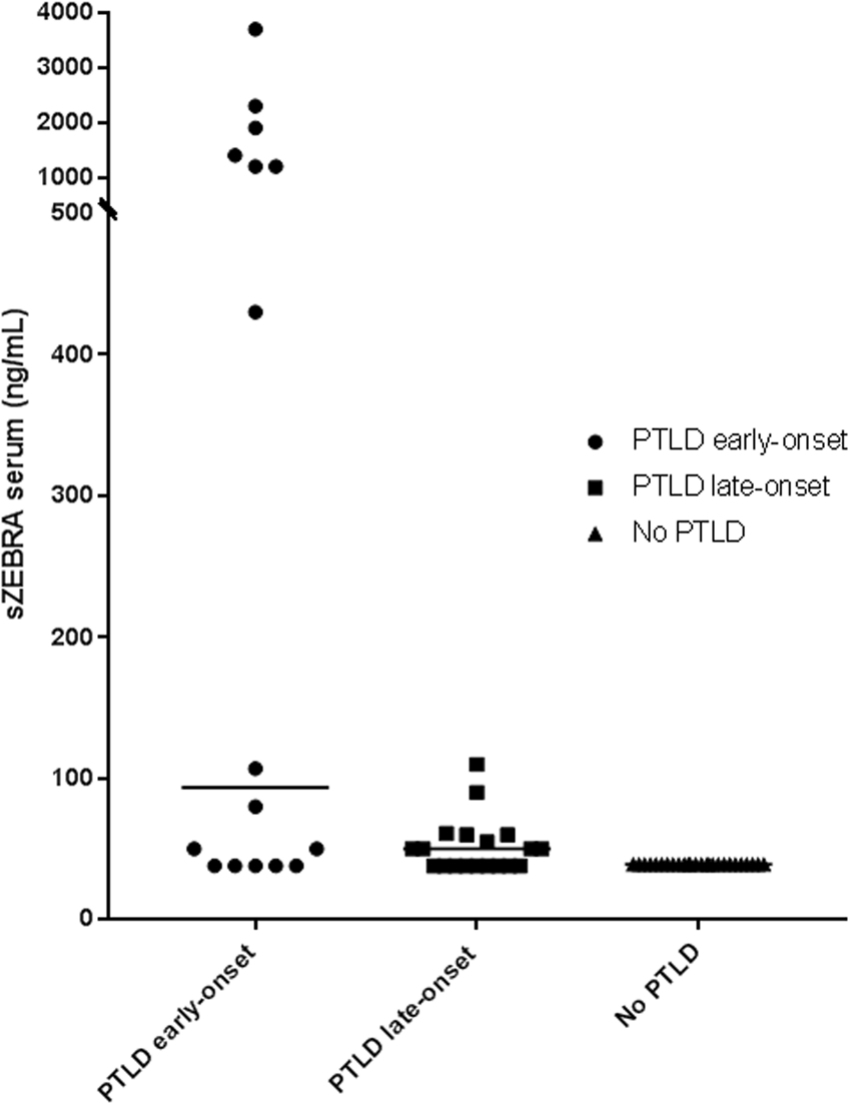
Quantification of sZEBRA at diagnosis of PTLD, in serum samples from patients with early-onset PTLD, late-onset PTLD. The LOD corresponds to 40ng/mL. The sZEBRA level was significantly higher in early-onset PTLD, compared to patients with late-onset PTLD, and the controls (p = 0.0056). The group called “no PTLD” includes the asymptomatic patients without any PTLD.

The EBV load and sZEBRA presence (>40ng/mL) at PTLD diagnosis have been provided in Table 2. EBV DNA (>200 copies/mL) and sZEBRA were detectable in 51% and 60% of cases, respectively. The positivity rates of sZEBRA and qPCR were very similar in the cases of EBV-positive B-cell lymphoma, at the time of diagnosis. The mean OD_450_ (+/− standard error of mean SEM) values of sZEBRA were significantly higher (p < 0.0045) in cases (0.870 +/− 0.200) than control patients (0.236 +/− 0.02) (Fig. 1A). Similarly, at diagnosis, the mean sZEBRA value in the cases, was 399 ng/mL +/− 141 *versus* 53ng/mL +/− 7 in the controls (p < 0.001) (Fig. 1B). Figure 2A shows the high correlation between OD_450_ values and sZEBRA levels (R^2^ = 0.828 p < 0.0001). However, there was no significant correlation between EBV load measured by qPCR and sZEBRA in all the patients enrolled (R^2^ = 0.107 p = 0.072) and in the PTLD patients (R^2^ = 0.0806, p = 0.0983)(Fig. 2B). Based on their clinico-pathological characteristics, we divided our patients in two groups according to the PTLD occurrence (16 patients with early-onset PTLD *versus* 19 patients with late onset PTLD)(Table 2). At diagnosis, the mean (+/− SEM) sZEBRA value in patients with early-onset PTLD was 787.9 +/− 271.7 ng/mL *versus* 51.47 +/− 4.46 ng/mL in the patients with late-onset PTLD (p= 0.0056). There was no significant difference between the sZEBRA values in late-onset PTLD patients and the controls (51.47 +/− 4.46 ng/mL *versus* 52.71 +/− 6.90 ng/mL, respectively) (p = 0.89) (Fig. 3). All the lymphomas occurring in HSC transplant patients were early-onset PTLD (12 out of 16). Interestingly, in the late-onset PTLD patients, sZEBRA was positive in four of the seven EBV-negative lymphomas, whereas PCR was negative in these last cases. sZEBRA was detectable in four of the nine cases of atypical PTLDs, whereas only one Hodgkin’s lymphoma patient was positive when using both PCR and sZEBRA at clinical diagnosis (Table 2).

### Retrospective follow-up of transplant patients

We retrospectively followed-up the sZEBRA-positive patients with multiple serum specimens available. The median follow-up was 8 months (range: 1 month −24 months). The follow-up panel in Figure S5 illustrates the kinetics of EBV load and sZEBRA in sera from patients prior the PTLD episode. In these selected patients, it is worth noticing the precocity of sZEBRA compared with EBV DNA (on average within 10 weeks preceding PTLD diagnosis).

## Discussion

This is the first report pertaining to the detection of a soluble form of the ZEBRA protein in serum specimens, as well as the first analysis on the replicative nature of EBV by using this putative new biomarker in PTLD patients. First of all, we focused on the development of an antigen-capture ELISA assay specifically designed to detecting the circulating ZEBRA protein. A similar *in vitro* approach had been performed to detect ZEBRA secreted by EBV-infected B- cells^30^, indicating it to be possible to explore a soluble form of ZEBRA in the extracellular milieu^28^. One interesting finding coming out from our study was that AZ125 and AZ130 mAbs, specifically directed against linear ZEBRA epitopes, were shown effective for capture and detection, respectively. The identity of these linear B-epitopes was defined by means of mAbs binding to a set of peptides, as previously reported^31^. Moreover, the amino-acid sequences of the identified peptide epitopes as recognized by the mAbs, corresponding to conserved EBV T-cell epitopes^32, 33^, were found to be highly specific to the ZEBRA protein. We thought that evaluation of the soluble circulating immediate-early DNA-binding protein ZEBRA (which transactivates lytic-cycle genes) in immunocompromised patients might contribute to new insights of EBV lytic cycle activation in these patients. With regard to EBV DNA levels, our results confirmed ealier studies investigating serum EBV DNA-emia monitoring in transplant recipients^34–37^. Detecting sZEBRA in the patients’ serum samples strongly suggests that: (i) during the EBV reactivation process, the protein is released from infected cells into the bloodstream; (ii) this protein possesses a sufficiently-long half-life to be efficiently detected in the patients’ serum. We thus suggest that ZEBRA would be released from either lysed tumor cells or through exosomes from unruptured EBV-infected cells^38^. It is worth noticing there are very few examples of non-structural viral proteins detectable in the serum of infected patients. This is the case with the HIV-1 *trans*-activator of transcription (Tat) being actively released from unruptured HIV-1-infected cells, thus detectable in *ex vivo* culture supernatants and in the serum of HIV-1 infected individuals at concentrations of up to 40ng/mL^39, 40^. The NS1 protein of the dengue virus is another good example of a non-structural protein, which is released into the extracellular milieu^41, 42^. Concerning the exogenous Tat, it is worth noting that it displays so-called “cell-penetrating peptides” (CPPs) being able to enter both uninfected and latently-infected cells^43, 44^, thereby inducing apoptosis in the former and activating the viral genome transcription in the latter. Interestingly, we demonstrated that ZEBRA exhibits the same cell-penetrating properties^45, 46^, like HIV-1 Tat^43, 47^, and therefore hypothesized that ZEBRA could be involved in EBV pathogenesis, not only as an essential protein for EBV replication activation but also as a “toxin” released in the extracellular milieu. All in all, we hypothesize that early abortive infection associated with fully lytic cycles may occur in the tumor or its environment, eventually releasing ZEBRA in the bloodstream. At the end, this phenomenon could stimulate the secretion of cytokines and factors promoting angiogenesis, B-cell proliferation, thereby further aggravating the immunosuppressive environment^25^, as we frequently detected sZEBRA associated with high serum IL-10 levels (data not shown).

Studies conducted on EBV lytic proteins (especially the immediate-early proteins like ZEBRA) in patients with PTLD are scarce and mostly related to the role of EBV proteins and gene products in neoplastic tissues^48–51^. It is important to point out that several authors exploring BZLF1 transcripts in peripheral blood lymphocytes (PBL) of PTLD patients demonstrated that both high EBV genome number and BZLF1 mRNA expression were sensitive markers of EBV-related PTLD^52^. In a previous study, we demonstrated ZEBRA expression in the whole PBMCs from a patient exhibiting a lymphoproliferative disease by means of flow cytometry. In this patient having undergone non-myeloablative allogeneic stem cell transplantation, the ZEBRA antigen was found in 4.85% of peripheral blood mononuclear cells^53^. In this patient, infected cells were found in the peripheral blood at higher levels (*e.g*. 1 to 10 lytic-infected cells per 10^4^ B lymphocytes^2, 9^ (*versus* 1 and 50 per 10^6^ B cells in persistently-infected healthy individuals^54^). It is interesting to note that the patients with early-onset PTLD have sZEBRA values significantly higher than the patients with late-onset PTLD. 6/16 patients with early-onset PTLD are HSC transplant patients, with very high sZEBRA values (mean value 1950 ng/mL), compared with patients with solid organ transplantation (mean value 67 ng/mL). We believe that our study may not definitely answer the question about these high values of circulating sZEBRA in HSCT patients. Further studies possibly using alternative markers of lytic state – are warranted to answer this question. Another important question could be the assessment of the exact kinetics of sZEBRA before PTLD’s occurrence. Since there are high titers of anti-ZEBRA antibodies in the serum of PTLD patients, another point will be to explore in the near future the possible existence of circulating immune complexes. Nevertheless, we guess the quantity of anti-ZEBRA IgGs in our population of PTLD patients have little influence on the sZEBRA detection efficiency. Indeed, in a majority of patients with sZEBRA  < 40 ng/mL, low or moderate titers of anti-ZEBRA IgG (range  < 1,000–5,000) were measured, so it’s unlikely that the absence of detectable sZEBRA would be due to a masking of the antigenic sites by host anti-ZEBRA IgGs.

Given that we failed to detect EBV DNA in the serum of all patients with EBV-negative B-cell lymphoma, sZEBRA detection proved particularly noticeable for this PTLD subtype and to a lesser extent for atypical PTLDs. The EBV-negative B-cell lymphomas represent a minority of the PTLDs^55^, and it was quite intriguing to detect sZEBRA in three patients out of seven. These findings could be accounted for by EBV replicating in the immune cells that make up the cellular environment in which the tumor cells reside, this was shown to significantly influence prognosis in different lymphoma subtypes^25^. We also hypothesize that early abortive replication associated with fully lytic cycles may occur in either the tumor or its environment. We failed to detect both sZEBRA and EBV DNA in the serum in 10 cases, namely kidney transplant patients with late-onset PTLD (8/10) and this shows a significant incongruity of EBV (both replicative and latent) and clinical course. It should be noted that authors have underlined the complex and heterogeneous relationship between PTLDs and EBV, as well as the lack of a close correlation between viral load and PTLD^56–58^ occurrence. Concerning patient follow-up, it is interesting to note that sZEBRA protein could be detected during periods in which EBV DNA-emia was not detectable when using qPCR. This discrepancy may be accounted for by the precocity of the ZEBRA signal measured over the course of EBV infection in this patient population. This precocity of sZEBRA detection when using qPCR is not dependent of the PCR format, since we observed the same phenomenon in PTLD patients investigated by means of EBV load measurement (expressed in copies/150,000 cells)(data not shown).

In this retrospective study, we reported for the first time the detection of soluble ZEBRA in serum of PTLD patients. These results showed that lytic EBV infection can be detected in what has been dogmatically believed to be latent-EBV driven B-cell replication. To summarize, it will relevant to investigate the lytic EBV infection in immunocompromised patients (such as organ transplant recipients) who are highly prone to the development of EBV -associated malignancies. We assume with respect to circulating ZEBRA: (i) it may be a marker of over-immunosuppression by triggering the expression of immunomodulating cytokines; (ii) it may consequently play a specific role in the oncogenic process.

## Electronic supplementary material

Supplementary information
