# Supplementary material for: Lytic EBV infection investigated by detection of Soluble Epstein-Barr virus ZEBRA in the serum of patients with PTLD
Source: Sci Rep. 2017 Sep 5;7:10479. doi: 10.1038/s41598-017-09798-7 (PMC5585268; doi:10.1038/s41598-017-09798-7)
Supplement: Supplementary file 1 — Supplementary information [file 41598_2017_9798_MOESM1_ESM.pdf]

Supporting Information for

Lytic EBV infection investigated by detection of Soluble Epstein-Barr virus ZEBRA in the serum of patients with PTLD

Mohammed Habib[1], Marlyse Buisson[1,2], Julien Lupo[2] , Felix Agbalika[3], Gérard Socié[4], Raphaele Germi[2], Monique Baccard[2], Berthe-Marie Imbert-Marcille[5], Jacques Dantal[6], Patrice Morand[2], and Emmanuel Drouet*[1]

Cloning and expression of the recombinant ZEBRA (r-ZEBRA) protein

Full-length r- ZEBRA plasmid pET15b (AS 1-245pET15b) was used to transform competent *Escherichia coli* BL21 (DE3), cultivated in Luria broth (LB) medium, supplemented with ampicillin at 37°C to an optical density (OD) of 0.6–0.8 at 600nm. Following 4 hours (h) of isopropyl β-D-1-thiogalactopyranoside (IPTG) induction at 37°C, the cells were harvested by centrifugation, suspended in 20mM Tris–HCl at pH 8.0 and 250mM NaCl (Tris–NaCl buffer) with 10% glycerol, then lysed by sonication. The resulting bacterial extract was treated with deoxyribonuclease I (DNase I) (Roche Applied Science) for nucleic acid removal. His_6-tagged proteins were then purified by means of nickel affinity chromatography, washed with a 0.5–1.5 M NaCl gradient and 20mM imidazole, and eluted in 500mM imidazole, 20mM Tris, 75mM KCl, 0.5mM NaCl, and 10% glycerol. All the purification steps were carried out at 4°C with protease inhibitors (complete protease inhibitor mixture; Roche Applied Science).

Purification of AZ125 and AZ130 monoclonal antibodies and labelling: These monoclonal antibodies (mAbs AZ125 and AZ130) have been obtained after immunization of

balb/c mice by the recombinant ZEBRA protein [60] and recognize respectively an epitope located in the N-terminal ZEBRA fragment (^{75}FSAPQPAPENAY^{86} , and one located in the C-terminal ZEBRA (^{184}VASRKCRAKFKQ^{195}) [60] [31]. The purification process of both mAbs was conducted by affinity chromatography on a protein A Sepharose column. Biotin labeling of AZ130 mAbs (IgG1 kappa) followed the method prescribed by the manufacturers (Pierce Protein Biology, Thermo Scientific, France). Briefly, the AZ130 mAb (2mg/mL) was dialysed against 1000mL of biotin labeling buffer (PBS, pH 7.4) at 4°C with three changes. The concentration of Sulfo-NHS-LC-Biotin was adjusted, in line with the manufacturer's instructions, and added to the appropriate volume of protein solution. The reaction was incubated for 3 hours at 4°C under gentle shaking. The excess biotin reagent was removed using a Thermo Scientific Zeba Spin Desalting Column.

Figure S1: Principle of the sZEBRA antigen-capture ELISA assay.

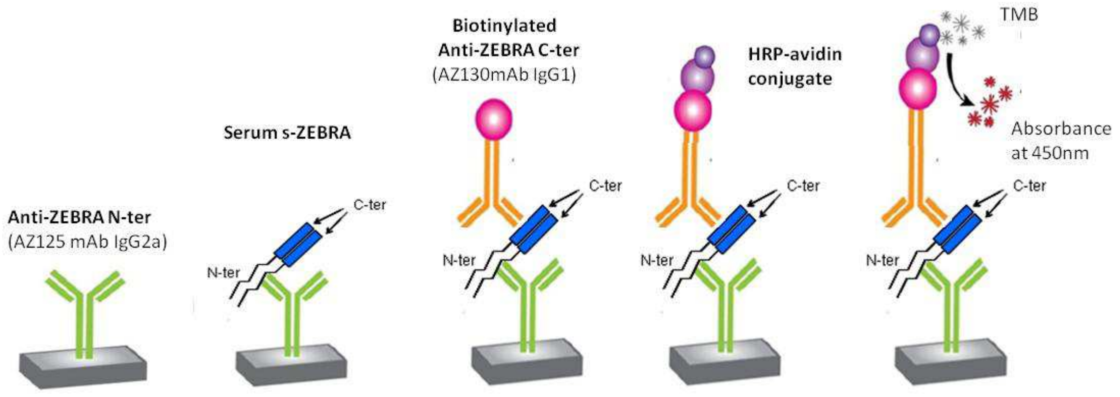

Figure S2: Measurement of sZEBRA by an antibody –based antigen-capture ELISA in the control population (30 EBV-seronegative subjects and 25 immunocompetent individuals with EBV serological reactivation)

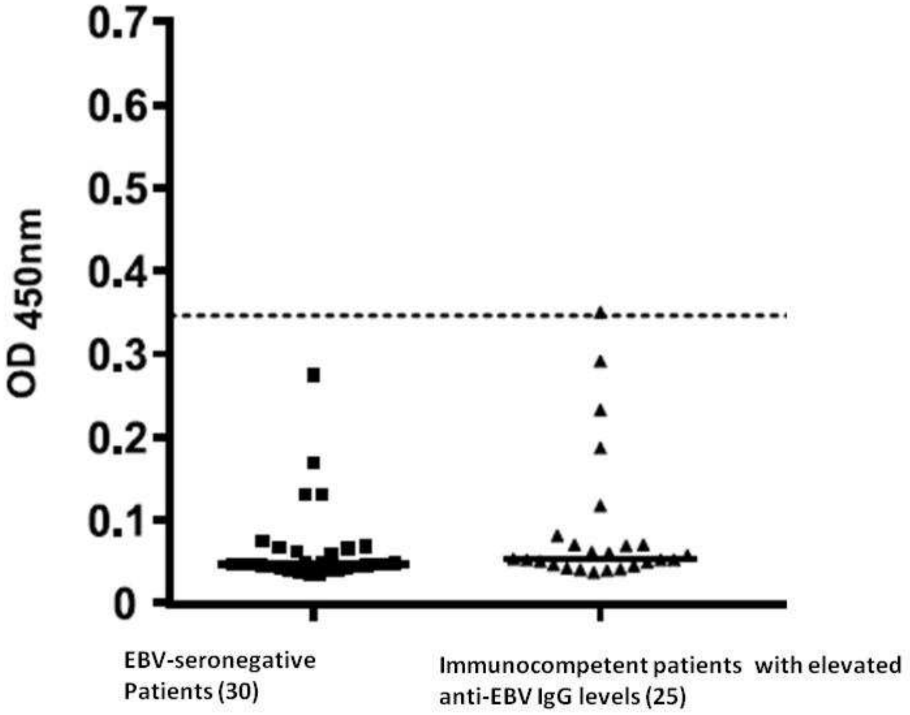

Figure S3: Evaluation of the specificity of the sZEBRA signals: We selected three positive serum samples (A = #11M B= #16A, and C = #13A,)(see table 2) (3,700ng/mL / 34,000 copies/ml - 2,300 ng/ml / 1,600 copies/ml - 1,900ng/ml / 5,000 copies/ml, respectively), and conducted a neutralization test by mixing each serum sample (1/10 dilution) with increasing concentrations of AZ130 mAb at a volume ratio of 1:1.

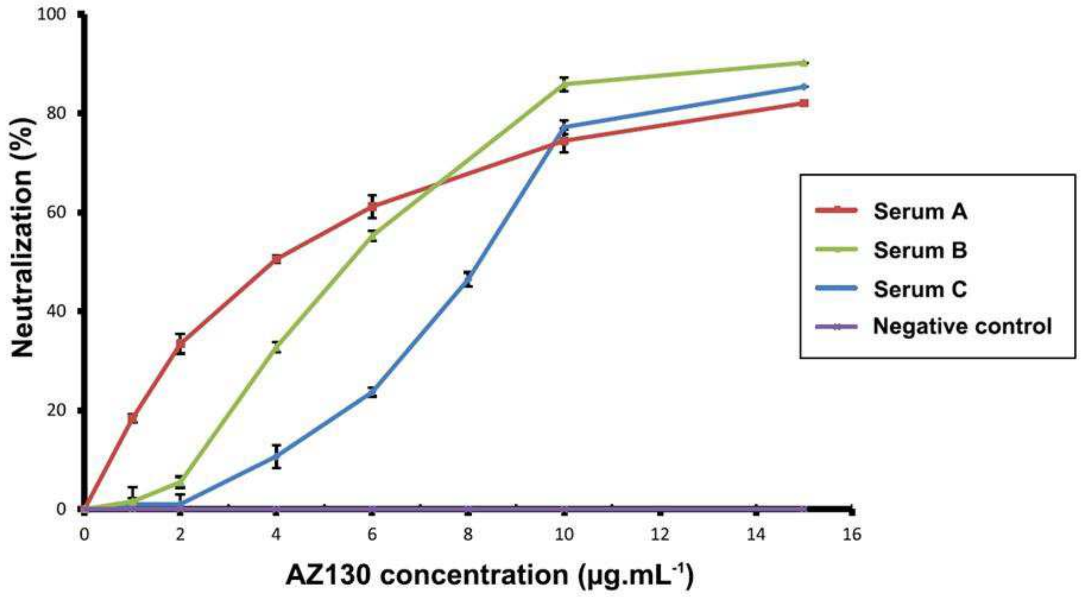

Figure S4: Development of the PTLD over time.

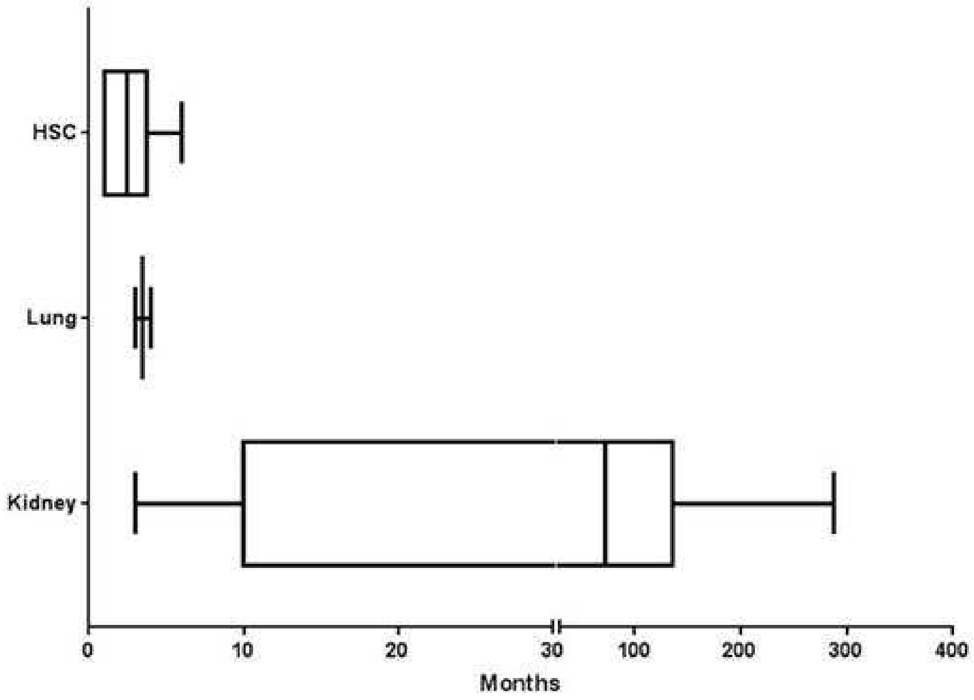

Figure S5: EBV load and sZEBRA quantification in four patients with PTLD: Serum samples were collected at diagnosis and at different time intervals before PTLD occurence, depending on the clinical course (sZEBRA in solid red line, EBV DNA load in dashed black line). The Patient 1 had positive signals (qPCR and sZEBRA) at PTLD diagnosis and demonstrated the situation of monitoring EBV in EBV-positive B-cell lymphoma with ever measuring sZEBRA in a period of more than 8 months. The patient 2 had also positive signals (qPCR, and sZEBRA) at PTLD diagnosis. The sZEBRA and EBV load values increased over time until the occurrence of a B-cell lymphoma. The patient 3 had a follow-up pattern similar to patient 2. A B-cell lymphoma (complicated with a GvHD) occurred after parallel increase of sZEBRA and PCR; then, a dramatic decline in sZEBRA and EBV load was observed after rituximab therapy until the patient unfortunately died. Patient 4 demonstrated a mantle lymphoma which was not associated with an increase in EBV load, although sZEBRA was positive earlier.

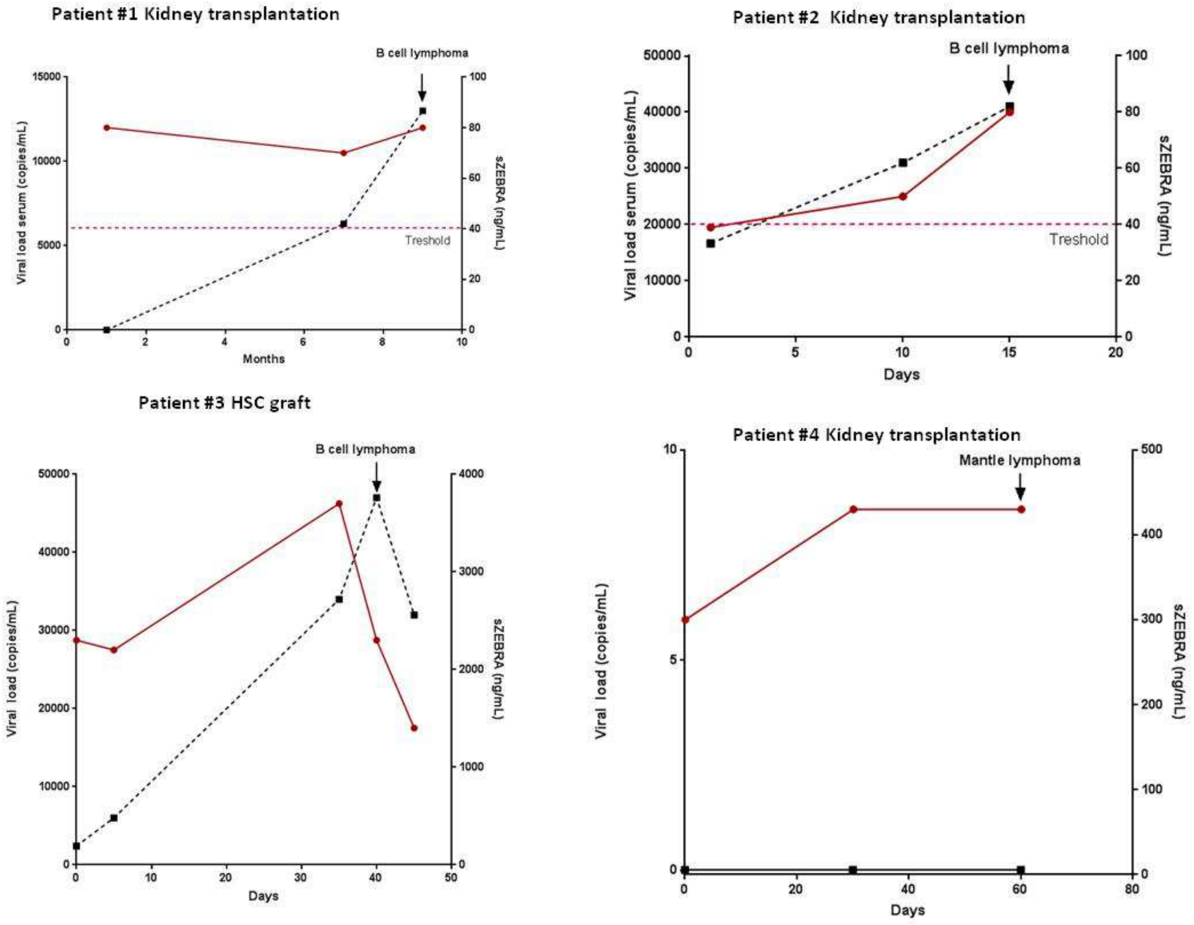

Supplementary References

60. Mikaelian, I. et al. The DNA-binding domain of two bZIP transcription factors, the Epstein-Barr virus switch gene product EB1 and Jun, is a bipartite nuclear targeting sequence. J Virol 67, 734–742 (1993).
